# Artificial intelligence in coronary artery calcium score: rationale, different approaches, and outcomes

**DOI:** 10.1007/s10554-024-03080-4

**Published:** 2024-05-03

**Authors:** Antonio G. Gennari, Alexia Rossi, Carlo N. De Cecco, Marly van Assen, Thomas Sartoretti, Andreas A. Giannopoulos, Moritz Schwyzer, Martin W. Huellner, Michael Messerli

**Affiliations:** 1 Department of Nuclear Medicine, University Hospital Zurich, Rämistrasse 100, Zurich, 8091 Switzerland; 2 University of Zurich, Zurich, Switzerland; 3 Division of Cardiothoracic Imaging, Department of Radiology and Imaging Sciences, Emory University, Atlanta, GA USA; 4 Translational Laboratory for Cardiothoracic Imaging and Artificial Intelligence, Emory University, Atlanta, GA USA

**Keywords:** Computed tomography, Coronary artery calcium, Coronary artery calcium score, Artificial intelligence, Machine learning, Deep-learning

## Abstract

Almost 35 years after its introduction, coronary artery calcium score (CACS) not only survived technological advances but became one of the cornerstones of contemporary cardiovascular imaging. Its simplicity and quantitative nature established it as one of the most robust approaches for atherosclerotic cardiovascular disease risk stratification in primary prevention and a powerful tool to guide therapeutic choices. Groundbreaking advances in computational models and computer power translated into a surge of artificial intelligence (AI)-based approaches directly or indirectly linked to CACS analysis. This review aims to provide essential knowledge on the AI-based techniques currently applied to CACS, setting the stage for a holistic analysis of the use of these techniques in coronary artery calcium imaging. While the focus of the review will be detailing the evidence, strengths, and limitations of end-to-end CACS algorithms in electrocardiography-gated and non-gated scans, the current role of deep-learning image reconstructions, segmentation techniques, and combined applications such as simultaneous coronary artery calcium and pulmonary nodule segmentation, will also be discussed.

## Introduction

 Atherosclerosis, a multifactorial, dynamic, heterogeneous disease, profoundly impacts morbidity and mortality, imposing a substantial economic burden on the healthcare system [1, 2]. This complex, inflammatory-based disease affects the elastic and muscular arteries leading to the formation of atherosclerotic plaques [3, 4]. Atherosclerotic plaques involve arteries’ intima and consist of a mixture of lipid, foam cells, debris, connective-tissue elements, and immune cells, inducing asymmetric focal thickenings of the affected vessels [3, 4]. There is a desire for simple, non-invasive biomarkers to quantify and characterize this disease, particularly in the cardiovascular field [5]. Calcium deposition and plaque calcification is a well-known process involving the lipid core of atherosclerotic plaques [5]. In patients with unknown atherosclerotic cardiovascular disease (ASCVD), coronary artery calcium (CAC) quantification, measured on computed tomography (CT) images (also named CAC score, CACS, or Agatston score), proved to be a robust, reliable and reproducible marker of subclinical coronary atherosclerosis [6–9]. CACS is a well-grounded approach for primary ASCVD risk stratification and a predictor for cause-specific cardiovascular mortality [6–9].

The groundbreaking advances of artificial intelligence (AI) in recent years and those foreseen in the future are expected to drastically change medicine, improving patient diagnosis, tailoring therapeutic strategies to patient needs, and relieving the medical community of tedious, error-prone tasks [10–12]. Imaging is one of the medical specialties at the forefront of the AI revolution. The ubiquitous presence of CT scanners [13], their short scan time, superiority (over other imaging techniques) in detecting vascular calcification [14], and the high clinical relevance of CAC detection [15], paved the way for CACS to embody the perfect task to benefit from the development and progress of AI. In fact, while plane chest X-ray, coronary angiography, ultrasound, and magnetic resonance, have the potential to image calcium deposition in the vessels, only CT can accurately quantify it [16].

In this critical review, we aim to describe the current state of research on this topic. Also, by introducing basic concepts of AI, we will unveil their potential, fully exploring the expected advances for healthcare and setting the ground for a thorough understanding of their use in CACS.

## Coronary artery calcium score (CACS)

In 1990, Agatston et al. provided pivotal evidence on the capability of (electron-beam) CT to quantify CAC, theorizing the CACS [17]. Since then, technological improvements in CT scanners have been tremendous, but the elegance and simplicity of the proposed method ensured CACS’s longevity. The Agatston score is a radiological construct based on 3 mm, electrocardiogram-gated (ECG-gated), non-contrast images, which relies on a semiautomatic quantification of calcified plaques, adopting 130 Hounsfield Units (HU) and 1 mm^2^ as the minimal cutoffs to differentiate calcified plaques from random noise [17]. Agatston score is the product of the weighted sum score of the peak density multiplied by the plaque’s area, summed across all eligible lesions (defined by n in the formula underneath) [18].

$${\text{Agaston}}\,\,{\text{score}} = \sum\limits_{{i = 1}}^{n} {\left( {{\text{Plaque's}}\,{\text{area}}_{i} \times {\text{Density}}\,{\text{weighted}}\,{\text{score}}_{i} } \right)}$$

Since its introduction, minimal changes have been made to the technical parameters for acquiring the CACS images. However, the introduction of multidetector CT, the most prominent change since CACS theorization [19], forced the standardization of the scanning protocol, which was achieved in 2007 owing to the work of McCollough et al. [20] (Table 1). Since then, imaging societies have discouraged any possible modification to these parameters [21].

**Table 1:** Technical parameters of CACS acquisition

Technical parameters	
Patient position	Supine
Type of acquisition	Axial
Scanning mode	Prospective, ECG-gated
Scan range	From below the aortic arch to the base of the heart
R-R interval	70-80%
Slice thickness	2.5-3 mm
Reconstruction thickness	2.5-3 mm
Peak tube voltage	120 kV
Tube current	Modulated current based on BMI
Reconstruction algorithm	Filtered back projection
Reconstructed matrix	512 X 512
Patient preparation	
Dietary preparation	Not needed
Pharmacological therapy	No changes
β-blockers	Not mandatory
Contrast medium	Not administered

*BMI* body-mass index, *CACS* coronary artery calcium score, *ECG* electrocardiogram, *mm* millimeter

The CACS can be expressed in absolute or relative values. The latter weighs the CACS according to the age-, sex-, and race-specific percentile [8]. Absolute values stratify the near-to-midterm ASCVD risk (5 to 10 years), whereas the relative score compares the ASCVD risk of the patient with that of peers [22]. While both methods can predict coronary events, absolute values had better performances and a more robust correlation with event risk [22]. In asymptomatic patients, CACS risk scores are grouped into the following categories: very low (CACS 0), mildly increased (CACS 1-99), moderately increased (CACS 100-299), moderately-to-severely increased (CACS 300-999), and severely increased (CACS ≥ 1000) [23, 24]. Since 2018 CACS reporting has been standardized according to the CAC data and reporting system (CAC-DRS) issued by the Society of Cardiovascular Computed Tomography (SCCT) [24].

Although the Agatston score is the most widely adopted method, it is not the only score to evaluate CAC. The calcium volume and calcium mass represent the total volume of calcified voxels and the “true mass” of calcium in the coronary tree, respectively [19, 25]. Both calcium volume and calcium mass had higher interscan reproducibility compared to the Agatston score [19, 26, 27]. Additionally, the results of a sub-study of the Multi-Ethnic Study of Atherosclerosis (MESA) on 3,398 participants with CACS > 0 showed the benefits of adding calcium volume and calcium density to coronary artery disease (CAD) and ASCVD risk evaluation. Interestingly, calcium volume was associated with a stepwise increase in CAD and ASCVD risk, while calcium density scores showed a stepwise decrease in the risks [19, 27]. These results helped disentangle the inverse relation existing between calcium density and ASCVD risk [6]. Indeed, highly dense plaques are more stable and inversely associated with ASCVD risk factors, begetting a lower ASCVD risk [19, 28, 29]. Therefore, calcium density improved CACS score based ASCVD risk quantification, solving some of the Agatston score’s intrinsic imperfections.

### Clinical value of CACS

Recently, several clinical guidelines endorsed the use of CACS to up or down-stratify asymptomatic, middle-aged patients at intermediate risk of ASCVD [15] and to guide therapeutic decisions (Table 2). Nonetheless, the benefits of CACS have not been fully exploited yet. Recent evidence showed that CACS-weighted pre-test probability models better stratified the risk of obstructive CAD compared to those based on conventional risk factors in patients with typical and atypical chest pain [30]. Additionally, by reclassifying 54% of patients to a lower CAD pre-test probability based on their CACS, it reduced downstream diagnostic testing [30].

**Table 2:** CAC use according to the suggestion from the current international guidelines

Guideline	Population	Role of CACS	Reference
American college of cardiology / American Heart Association	Adults (40 to 75 years of age) at intermediate (7.5-20%) or selected adults at borderline (5-7.5%) 10-year ASCVD risk with uncertain risk-based decision for preventive therapy	CACS = 0 consider withhold statin (unless diabetes, smoking, etc.) CACS 1-99 favor statin (particularly, >55 years of age) CACS >100 or >75^th^ percentile start statin therapy	[116]
	Younger or older adults (<45 or ≥75 years of age, respectively), women at lower 10-year ASCVD risk (<7.5%), or selected low-risk adults (<5%) 10-year ASCVD risk	Refine ASCVD risk	
Canadian Cardiovascular Society^$^	Adults (>40 years of age) at intermediate (10-19.9%) 10-year FRS risk with an uncertain risk-based decision for preventive therapy	Initiate statin therapy if CACS >0	[117]
	Selected adults (>40 years of age) at low (<10%) 10-year FRS risk (familiar history of premature ASCVD)	Initiate statin therapy if CACS >0	
European Society for Cardiology / European Atherosclerosis Society	Asymptomatic individuals at low or moderate ASCVD risk who are eligible for statin therapy or those who were not able to lower cholesterol levels with lifestyle intervention alone	CACS >100 determines upward risk reclassification, leading to considering statins use	[118]
United Kingdom National Institute for Health and Care Excellence	Asymptomatic patients with suggested electrocardiographic changes for ischemia	Adjudication statin allocation	[15]
Cardiac Society of Australia and New Zealand^%^	Asymptomatic adults (45 to 75 years of age) at intermediate risk score (10-20%) for 10-year ASCVD	CACS = 0 no treatment CACS 1-100 improve diet and lifestyle changes CACS 100-400 aspirin recommended; statin considered reasonable CACS >400 statin and aspirin recommended	[119]
	Patients at lower 10-year risk (6-10%) with a strong family history of premature ASCVD or diabetic patients aged 40 to 60 years old		
Japanese Atherosclerosis Society^+^	Not included in the predictive model. However, it is regarded as a prognostic tool in intermediate-to-high-risk individuals	Further high-quality studies, specifically addressing the Japanese population, are needed	[120]
Chinese Society of Cardiology^&^	Not included in the predictive model. However, it may be used as a cardiovascular disease risk enhancement factor in patients aged 40 to 70 years of age who are at very high 10-year ASCVD risk	No role for CACS = 0 CACS ≥ 100 may trigger low-dose aspirin administration	[121]
National Lipid Association	Adults (40 to 75 years of age) at borderline- to intermediate-risk (LDL-C 70 to 189 mg/dL and 5-19.9% 10-year ASCVD risk)	CACS = 0 defer statin CACS 1-99 favor statin CACS 100-299 favor statin and aspirin CACS ≥ 300 high-intensity statins and aspirin	[122]
	Adults (40 to 75 years of age) with LDL-C 70 to 189 mg/dL and <5% 10-year ASCVD risk and family history of premature ASCVD	CACS = 0 lifestyle changes CACS >0 lifestyle changes and consider statins	
Society of Cardiovascular Computed Tomography	Adults (40 to 75 years of age) with a 5% to 20% 10-year ASCVD risk group	CACS = 0 statin not recommended CACS 1-99 moderate or moderate to high intensity statin CACS 100-299 moderate to high intensity statin and aspirin CACS >300 high intensity statin and aspirin	[23]
	Adults (40 to 75 years of age) with a <5% 10-year ASCVD risk who seek reassurance (ex. strong family history of premature CAD, etc.)	CACS = 0 or low values confirm the low-risk status Higher CACS identify individuals in whom lifestyle recommendations should be enhanced or treatment considered	

^$^: Clinical risk score is based on the Framingham Risk Score (FRS); ^%^: Clinical risk score is based on a tool generated by the National Vascular Disease Prevention Alliance based on the FRS; ^+^: Clinical risk score is based on the Suita score; ^&^: Clinical risk score is based on the risk assessment algorithm for primary prevention of cardiovascular disease in Chinese adults; *ASCVD* Atherosclerotic cardiovascular disease, *CACS* Coronary artery calcium score, *CAD* coronary artery disease, dL deciliter, *FRS* Framingham Risk Score, mg milligram

Of note, while the “*power of 0*” is still debated [31], the 10-year risk of all-cause mortality of CACS 0 is < 1% [19]. Conversely, minimal increases (CACS 1-10) are associated with a two-fold increase in the overall mortality rates [32]. Moreover, individuals with a CACS ≥ 1000 have five times the risk of ASCVD and roughly three times the risk for all-cause mortality of those with a CACS 0 after adjusting for conventional risk factors [33]. Even more alarming is the absence, of a disease-specific and all-cause mortality plateau effect in this patient population [33].

### CACS in non-ECG-gated images

Although CACS was developed on ECG-gated images, a meta-analysis of 661 adults showed a high correlation between scores derived from ECG-gated and non-gated, non-contrast images [34]. This may improve ASCVD risk assessment in specific cohorts of patients, namely candidates for lung cancer (LC) screening and oncologic patients sharing risk factors with patients with ASCVD. Former or active smokers enrolled in the control arm of an LC screening randomized controlled trial had equivalent 10-year mortality rates of LC and ASCVD (24% and 21%, respectively) [35]. Indeed, older age and a heavier smoking history increased the likelihood of undergoing LC screening in a population-based study involving more than 14,500 adults, of whom 67% were overweight or obese and 22% had diabetes [36]. Further, 62% of patients enrolled in a cross-sectional LC screening study were CACS positive, 7% had values > 1000, and 57% of those qualifying for primary prevention statin therapy were not actively treated [37]. Tailor et al. corroborated this evidence, confirming that a minority of LC screening patients carried a diagnosis of ASCVD (31%), while most (74%) were eligible for statin therapy but not under active treatment [38].

However, performing CACS on non-gated images may underestimate the Agatston score. Xie et al. showed that 9% of CACS-positive and 19% of CACS ≥ 400 patients on ECG-gated images were downgraded to CACS 0 or < 400 on non-gated images, respectively [34]. A possible solution to the problem of accurate CACS quantification in non-gated images could be qualitative analysis. CAC-DRS also supports the qualitative evaluation of the presence and extent of coronary tree calcification on non-gated images [24, 39]. Qualitative CAC-DRS are divided into the following categories: very low (CAC-DRS 0), mildly increased (CAC-DRS 1), moderately increased (CAC-DRS 2), and moderate to severely increased (CAC-DRS 3), mirroring their quantitative counterpart, and providing similar ASCVD risk stratification and treatment recommendations [24].

### Limits and future directions of CACS

Neither the Agatston score nor CAC-DRS considers plaque location. However, involvement of the left main trunk and left anterior descending artery (LAD) is associated with a worse outcome [40–42]. Additionally, the higher the number of affected vessels, the higher the risk of CAD [43]. Hence, the SCCT suggested reporting CAC location, irrespective of the evaluation method applied [24]. Scan-rescan studies showed the complexity of measuring a modifiable parameter. Those with a short interscan period (23 ± 27 days) demonstrated low measurement variations [44]. Conversely, a sub-study of the MESA with an interscan period between 1 and 5 years showed that baseline CACS values, body mass index, and scanner factors must be considered when quantifying CACS variations in longitudinal studies [45]. Of the 2,832 patients with CACS > 0 at baseline, 85% showed an increase in CACS at follow-up scans [45]. However, 52% of the variation was attributable to the previous factors, and factor-adjusted analysis demonstrated that only 32% of the initial patients had increased CACS values [45]. An additional point not yet taken into consideration is the difference in CAC profiles between sexes. Women have fewer calcified plaques and less calcified vessels, ultimately generating a lower CAC volume [46]. Additionally, the proportion of women with detectable CAC typically rises at the age of 46, nearly a decade after that observed in men [46]. Nonetheless, CACS-positive women have an overall 1,3-fold higher relative risk of ASCVD-related death compared to men. This relationship increases with CACS values, being ~ 1,8-fold higher in women with CACS > 100 [46]. Interestingly, a sub-study of the MESA involving 2,456 postmenopausal women showed that those with early menopause had a lower prevalence of CACS 0 (55%) than those without early menopause (60%) [47]. Further, in the coronary artery risk development in young adults study, the prevalence of women with CACS > 0 was 18%, 21%, and 13% for premature menopause, menopause ≥ 40 years, and premenopausal, respectively [48]. Of note, the results of these studies did not reach statistical significance, leaving the association between CACS and menopausal status unanswered. Finally, it is worth noting that, irrespective of sex, ~ 10% of CACS-negative individuals have non-calcified plaques, 1% have obstructive non-calcified plaques, and ~ 0.5% develop cardiac events at long-term follow-up (≥ 42 months) [49].

Installation and availability of dual-energy and photon-counting CT (PCCT) brought exciting perspectives into CACS [39, 50]. These techniques allow the reconstruction of virtual non-contrast (VNC) images from contrast-enhanced exams [18]. Therefore, CACS could be quantified from contrast-enhanced scans. However, VNC images proportionally underestimated CACS values derived from non-contrast images primarily because of an underestimation of plaque volume and density, requiring ad-hoc conversion factors to yield accurate results [51, 52]. Additionally, ultra-low dose non-enhanced CACS images acquired using a dual-energy scanner underestimated CACS-based risk categories in 17% of patients compared to their standard-dose counterparts [39]. PCCT, a pioneering technique directly converting the energy of X-ray photons into an electric pulse, can increase the contrast-to-noise ratio (CNR) and detect smaller and less calcified objects [50, 53]. PCCT scanners implemented in clinical routine have detectors of 2 mm thickness and a ~ 200 μm pixel dimension at the isocenter [54], lower than the ~ 1000 μm of energy integrating detectors [55]. While the foreseeable benefits of PCCT adoption are huge, studies comparing CACS between normal (energy integrating) scanners and PCCT showed a systematic reduction in CACS values in PCCT, leading to the reclassification of 5% of patients [56].

## Artificial intelligence

**Fig. 1:**
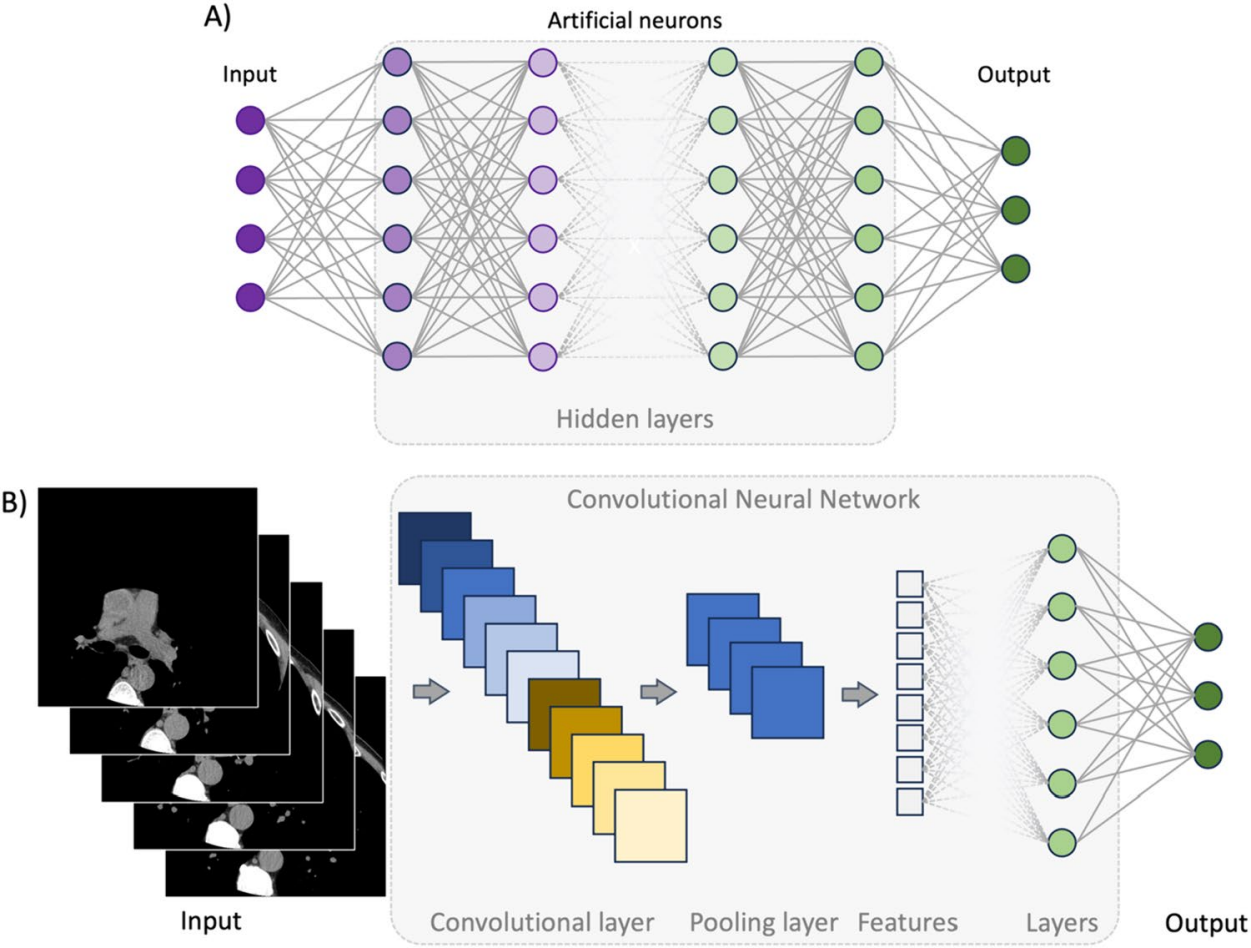
Deep-learning and convolutional neural network algorithm architecture. **A** Deep learning (DL) relies on multiple hidden layers of artificial neural networks (ANN), hence the name "deep". These layers are usually defined as "hidden" because they do not belong either to the input or output layer. The number of hidden layers determines the depth of a model. Their role is to capture patterns and features, transforming input data into other data forms usable by the subsequent layer of neurons. Indeed, each neuron contained in these layers relates to the formers. Information flows from layer to layer, moving from input to output, progressively increasing its complexity and abstractedness. **B** Convolutional neural networks (CNN) are the most common DL architecture used in image analysis. This architecture has two major components: *convolutional* and *pooling layers*. The former is the core building of a CNN and works by applying filters to the input data, generating an activation map. Pooling layers combine the outputs of the convolutional step, reducing the number of features extracted. These steps can be repeated multiple times. Usually, the last step is allocated to layers of artificial neural networks, which in turn generates the output. *ANN* artificial neural network, *CNN* Convolutional neural network, *DL* deep learning

AI can be defined as the creation and development of hardware and software capable of performing tasks usually confined to human intelligence or broadly as intelligence exhibited by machines [57, 58]. The implementation of AI is expected to improve medicine thanks to its ability to learn and adapt to a huge amount of data and handle onerous tasks requiring great cognitive dexterity [58–60]. Furthermore, it is expected to free clinicians from tedious and repetitive tasks still demanding undistracted attention [58–60]. At its core, AI encompasses a broad range of concepts, including but not limited to machine learning (ML), deep learning (DL), and convolutional neural networks (CNNs) (Fig. 1). Each of these concepts has a characteristic range of applications, is associated with distinct algorithm architectures, and has various layers of complexity.

ML learns patterns directly from data/examples [57, 59]. This overcomes the traditional rule-based approach that sees computers accurately reproducing the programmer’s instructions [59]. Several different components need to be considered when building an ML algorithm. Although a detailed description is beyond the scope of this review, we will briefly guide the readers through some ML approaches [61]. ML usually relies upon one of the following approaches: *supervised learning*, *semi-supervised learning*, *reinforcement learning*, or *unsupervised learning*. *Supervised learning* uses labeled datasets with a known output or outcome variable [57]. The model learns patterns and relationships existing between inputs (in our case images) and outputs by minimizing the difference between its predictions and the real outputs throughout an iterative, optimized process [57]. *Semi-supervised learning* overcomes the challenges often caused by the scarce availability of high-quality datasets curated by experts [57, 59]. This approach trains the algorithms using both labeled and unlabeled output data. *Unsupervised learning* opts for using the ML algorithm against unlabeled output datasets, mainly aiming to discover hidden patterns or structures within the data. This strategy relies on a naïve approach to data, exempting the algorithm from previous evidence and empowering it to unveil otherwise hidden connections and associations. Finally, *reinforcement learning*, infrequently used in medicine, finds a balance between exploration and exploitation in a specific environment by yielding different grades of rewards.

DL represents a subset of ML that uses stacks of artificial neural networks (ANNs) processing layers to perform representation learning on structured and unstructured raw data (Fig. 1) [62–64]. ANNs are inspired by the human brain, particularly the primary visual cortex, and are composed of interconnected linear units called neurons [62, 63]. As their biological counterpart, artificial neurons receive and process signals (using a non-linear activation function, mostly ReLU), activating (i.e., passing the information to subsequent units) based on the weighted sum of their inputs [65]. Artificial neurons have full pairwise connections with the following layers [65, 66]. Conversely, they are not connected to neurons of the same layer [65, 66]. Passing through multiple, hidden ANN layers extracting and transforming lower-level features into higher-level features, the input information is interpreted [57, 63]. Therefore, ANNs can learn different intricate representations at different levels of abstraction [62].

CNNs are a specialized type of ANN architecture, working best on grid-like topology data, such as images and videos. Therefore, they are commonly used to analyze medical images [62, 63]. Three layers form the CNN: convolutional, pooling, and fully connected layers (Fig. 1) [67]. The convolutional layer plays a vital role in CNNs by processing small regions of space of the input images using learnable filters, extracting local patterns and spatial relationships, and generating feature maps [67, 68]. Pooling layers down sample feature maps, preserving important information [68]. The output of the pooling layer is finally fed to fully connected layers of ANNs. A significant upgrade in CNNs came with the development of the so-called U-net and ResNet/VGGNet, which currently represent two of the most used algorithms for image analysis [65, 66]. U-net consists of a symmetric architecture, including a contracting and an expansive path, yielding a U-shaped appearance, and is largely employed for image segmentation [66, 69]. This architecture, devoid of any fully connected layers, relies only on convolutions [69], improving the spatial localization of image features and maintaining high performances in image classification. ResNet and VGGNet are classically adopted in image classification tasks. VGGNet was developed to increase the CNN depth by applying small-size filters [66], while ResNet overcame the degradation problem encountered as the depth increased [70, 71]. A detailed description of these networks’ architecture is beyond the scope of this review. However, further details can be found in the review from Alzubaidi et al. [66].

### AI performance assessment

**Fig. 2:**
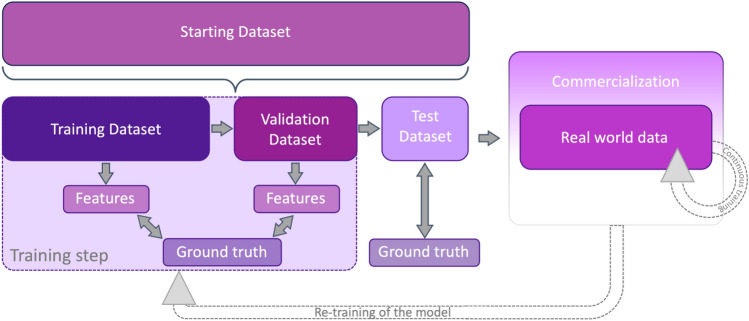
Steps required to create, validate, and commercialize an AI algorithm. AI algorithm/model creation always starts by identifying a research/clinical question, which dictates the starting dataset. Similarly, the algorithm’s architecture is selected among those performing best according to the data type. Subsequently, the starting dataset is subdivided into separate datasets of different dimensions, naming *training dataset*, *validation dataset*, and *test dataset*. The latter does not need to be generated from the starting dataset. Indeed, it is preferable to have an external test dataset. During the training step, the model analyzes the *training dataset,* deriving features that are tested against the ground truth. The identical process is performed on a separate dataset, the *validation dataset.* This validates the performances of the algorithm and fine-tunes it. Subsequently, the algorithm is tested on an additional separate dataset (the *test dataset*), and its final performances are evaluated. Valuable AI models are finally commercialized. After commercialization, the algorithms learn continuously from real-world data. Also, the model can be re-trained to overcome some flaws encountered when dealing with a real-world scenario. *AI* artificial intelligence

Datasets, model fitting, and model performances are crucial notions to understand the complexities and nuances of ML, DL, and CNNs. The *training dataset* is used to build the model/algorithm, whereas the *validation dataset* serves to tune and improve the learning performance of the algorithm and the *testing dataset* to evaluate the model’s performance (Fig. 2, i.e., accuracy, precision, recall, etc.), respectively [61, 62]. It is not surprising that the size of the *training dataset* should be tailored to the underlying task, with more complex tasks requiring larger datasets. Larger training datasets also obviate false pattern recognition due to imbalances in variables (i.e., sex, age, smoking status, etc.) used to build the algorithm [58]. *Training*, *validation*, and *testing datasets* need to be independent, with no overlap. Further, to increase the algorithm’s generalizability, the *testing dataset* should, ideally, be external [61]. These steps help reduce overfitting, which refers to an algorithm working exceptionally well on the data (images) it was trained on (*training dataset*) but then fails to generalize adequately to new, unseen data (*testing dataset*) [61].

Several performance metrics can be used to interpret the quality and accuracy of the results of AI algorithms. Accuracy evaluates the number of correct predictions within the whole dataset. Although useful, accuracy is generic and unable to disentangle the performance in different classes (i.e., sensitivity and specificity) [72]. The confusion matrix and the area under the receiver-operating curve improve the understanding of the performance of the classifier [72], while the F1-score evaluates the harmonic average between recall and precision rates [66]. Jaccard index and DICE similarity index are commonly used to grade image segmentation, measuring the degree of proximity between two segmentations on a pixel-wise analysis [72, 73]. Their values always lie between 0 and 1, with the two extremes representing the lack of or the exact correspondence between the segmentation generated by the algorithm and the ground truth [73]. Finally, signal-to-noise ratio (SNR), CNR, modulation transfer function, and noise power spectrum are commonly used to quantify the image quality. The mathematical formulas used to calculate these metrics are reported in Table 3.

**Table 3:** Evaluation metrics

Metrics	Formula
Accuracy	$\frac{{TP + TN}}{{TP + TN + FP + FN}}$
Sensitivity (or recall)	$\frac{{TP}}{{TP + FN}}$
Specificity	$\frac{{TN}}{{FP + TN}}$
Precision	$\frac{{TP}}{{TP + TN}}$
F1 score	$2 \times \frac{{{\text{Precision}} \times {\text{Recall}}}}{{{\text{Precision}} + {\text{Recall}}}}$
Area under the receiving operator	$\frac{{S_{p} - \frac{{n_{p} \left( {n_{n} + 1} \right)}}{2}}}{{n_{p} n_{n} }}$
Jaccard index	$\frac{{\left| {X \cap Y} \right|}}{{\left| X \right| + \left| Y \right| + \left| {X \cap Y} \right|}}$
DICE score	$2\frac{{\left| {X \cap Y} \right|}}{{\left| X \right| + \left| Y \right|}}$

*FN* false negative, *FP* false positive, *TN* true negative, *TP* true positive, *S*_*p*_ sum of all positive ranked samples, *n*_*p*_ number of negative samples, *n*_*p*_ number of positive samples, |X| and |Y|: representing the X and Y segmentations, respectively; |X∩Y|: representing the intersection between X and Y

## Translating AI concepts into CACS

### CAC detection and segmentation

**Fig. 3:**
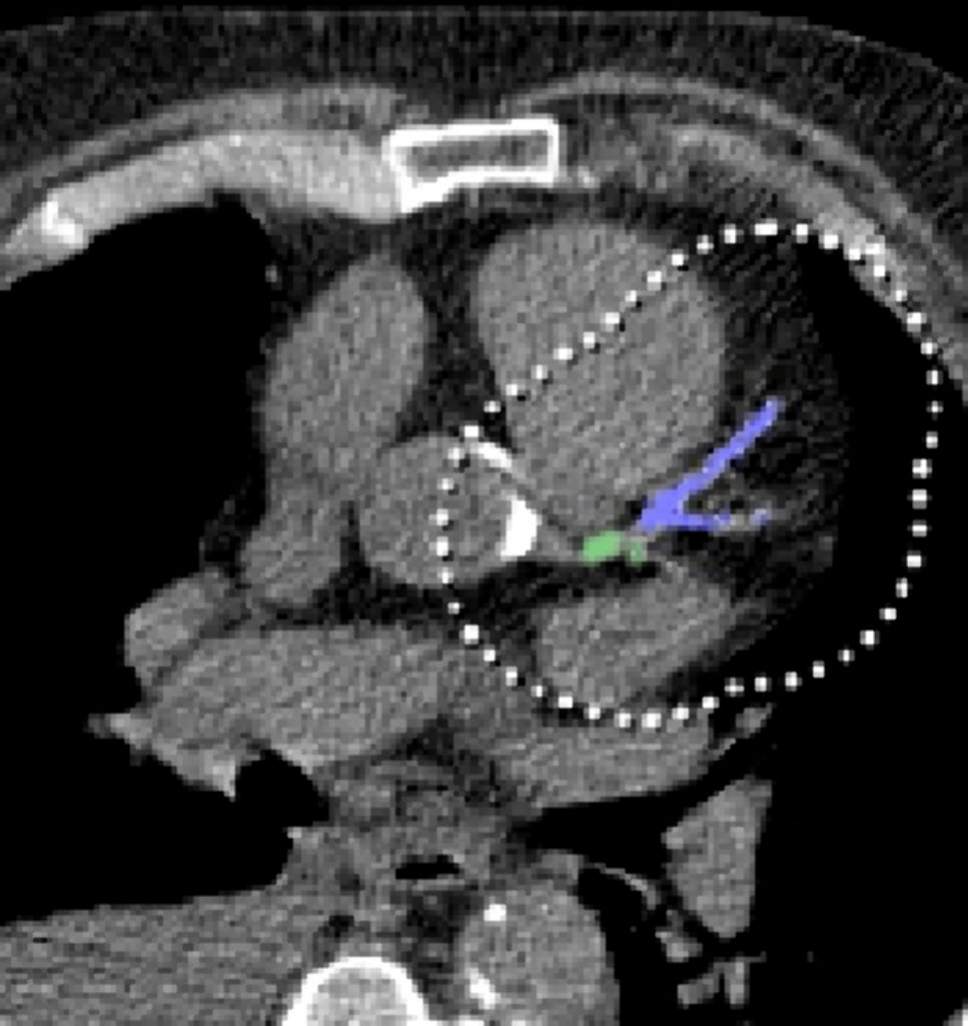
Coronary artery calcium segmentation. Automatic detection, segmentation, and classification of coronary artery calcium in the left main (light green) and left anterior descending artery (purple blue) in an 80-year-old man with severe coronary artery calcification (i.e., Agatston score: 2187).

CAC detection and segmentation strategies rely either on the intrinsic high density of calcified plaques or on the anatomic location of coronary arteries (Fig. 3) [74]. While these approaches seem robust, the presence of high-density cardiac (i.e., aortic and valvular calcification) and non-cardiac (i.e., lymph nodes, metal structures, noise, etc.) mediastinal structures, as well as the low coronary arteries-to-myocardium contrast difference on non-contrast images, are inherent difficulties encountered in automatic CAC segmentation algorithms creation [75, 76]. To date, the following approaches have been used in ECG-gated images:

- *Calcified plaque detection based on the localization of large structures* (i.e., cardiac profile and the aortic root). This approach allowed either image co-registration with previously built atlases, deriving the expected location of coronary arteries [77], or isolating the heart by applying various subsequent segmentation steps. Subsequently, calcifications were identified on the segmented images by image thresholding or geometrical constraints locating coronary arteries’ origin [78, 79].
- *ML-based selection of imaging features correctly classifying the presence of CAC* [75, 76]. In this scenario, different approaches were explored, ranging from those necessitating user inputs [75, 80] to fully automatized ones [76]. At their core, these approaches rely on letting software grow regions of interest from which different features were derived. Features were further subdivided into intensity-based features (i.e., mean or maximum density), spatial features (i.e., the cartesian coordinates of the plaque), or geometrical features (i.e., the shape and size of the plaque) [81]. The best feature combination, enabling accurate CAC detection, was calculated by combining and testing various models [75, 76, 80]. Interestingly, lesion location and plaque highest density always led to the best model performances, whereas shape- and dimension-related features were consistently discharged [75, 76]. The results of these approaches varied considerably, with a study reporting a calcification detection of ~ 74% [76], while others had sensitivity and specificity values of 92–93% and 98–99%, respectively [75].
- *ML-based derivation of imaging features obtained using coronary arteries-based atlases, created upon CT-angiography images* [74, 81, 82]. This method summed up the advantages of the formers using both lesion features and atlases and yielded CAC detection sensitivity between 81 and 87%, while specificity varied between 97% and 100% [82].

In non-gated CT images, the cardiac motion artifacts preclude the possibility of applying segmentation-based approaches [74]. Lessemann et al. overcame this shortcoming by proposing a two-stage CNN approach [74]. The first CNN’s large receptive field applied image thresholding, categorizing all voxels exceeding 130 HU and subdividing them according to the presumed coronary artery they belonged to, while the second CNN’s smaller receptive field refined the results of the previous subdividing authentic calcification from other high-density structures (false positive voxels) [74].

### CACS quantification

Fully automated CACS algorithms offered high performances on both ECG-gated and non-gated scans. In ECG-gated images, the mean difference between manually and AI-calculated Agatston scores was − 2.86 to 3.24 [83] in older algorithms and 0.0 to 1.3 in more recent ones [84]. Additionally, experts-to-AI intraclass correlation coefficient (ICC) values ranging from 0.84 to 0.99 were reported by several authors [84–88], while the expert-to-expert ones were 0.84, 0.85, and 0.77 [84]. Interestingly, the availability of additional datasets rather than adopting multiple models (in the same dataset) improved the AI-based accuracy [89]. While these results already proved the potential of AI, the adoption of U-net++, an ameliorated version of U-net [90], reduced the CACS error from 5.5 to 0.48, indicating a bright future for this application [91]. On non-gated images, ICC values of 0.99 and 0.90 were reported using chest CT scans [92] and low-dose chest CT scans acquired for LC screening [93], respectively. Similarly, AI and expert CACS evaluation had a good correlation on Bland-Altmann plots using non-gated chest scans [94]. However, the agreement of risk categorization between AI and expert evaluation varied according to the test dataset used, being between *k* = 0.58 and *k* = 0.80 on routine chest CT using external datasets [83] and between *k* = 0.85 [93] and *k* = 0.91 [74] on low-dose chest CT using internal ones. Similarly, the agreement between risk categories based on ECG-gated and non-gated images ranked from k = 0.52 to 0.82, with lower values obtained with external datasets [83, 95]. This proves the important connection between the algorithm’s performance and the dataset-specific characteristics (i.e., scanner, field-of-view, reconstruction filter, slice thickness, etc.) in non-gated images, resulting in a rapid performance degradation varying the input data [96]. In the study by Lessmann et al., the overall sensitivity of the algorithm, trained on soft reconstruction kernels only, decreased from 91 to 54% when applied to images reconstructed with a sharp kernel [74]. To overcome these issues, van Velzen et al. retrained their algorithm with a small additional set of representative data-specific examinations [96]. Supplementing the network with data-specific scans generated narrower confidence intervals (CIs) on Bland-Altman plots, indicating an improved correlation between the Agatston values calculated by the software and the reference standard [96]. Further, the combined reliability of risk category assignment improved from *k* = 0.90 to 0.91 [96]. However, the amount of supplementary data-specific scans needed to ensure optimal performance is unknown. Besides these drawbacks, a recent study on 5,678 adults without known ASCVD transitioned AI usage from research into a clinical scenario by utilizing a DL-based algorithm to analyze CACS on non-gated images, showing that adults with DL-CACS ≥ 100 had an increased risk of death (adjusted hazard ratio: 1.51; 95% CI: 1.28 to 1.79) compared to those with DL-CACS 0 [97]. A fascinating, additional perspective was provided by a cloud-based DL CACS evaluator showing high ICC value (0.88; 95% CI: 0.83 to 0.92) between ECG-gated and non-gated images derived from 18 F-fluorodeoxyglucose positron emission tomography (PET) [95]. Although these results were not replicated in a similar setting [98], cloud-based tools have the potential to broaden the users of AI-based CACS evaluation beyond university and tertiary hospitals, helping to reach its full potential.

Total CACS is currently used by international guidelines to guide therapeutic decisions; however, it is worth noting that CAC is unevenly distributed, mostly located in the LAD or the right coronary artery [41]. However, as previously discussed, heavily calcified left main trunk or LAD correlates with a higher mortality risk [41, 42]. Therefore, detailing algorithm performances on a vessel-based level is of uttermost importance but often overlooked or done by using dissimilar metrics, as described in Table 4.

**Table 4:** CACS values in the different branches of the coronary tree

First Author	Year of publication and reference	Structure of the algorithm	Dataset - Type of images	Number of patients	Age of patients in years, mean ± SD (or age range)	Performance				
						Metric	LM	LAD	LCx	RCA
Winkel *et al.*	2022 [84]	3-D U-net	ECG-gated CT	1171	56±10 (510 pts) 58±9 (399 pts) 60±10 (262 pts)	Accuracy	89%	91%	93%	100%
Hong *et al.*	2022 [91]	U-net++	ECG-gated CT	1811	58 (18 to 96*)	Detection rate	80%	97%	89%	94%
Zhang *et al.*	2021 [86]	3-D U-net	ECG-gated CT	232	55±13	ICC	0.98	0.99	0.97	0.98
Morf *et al.*	2022 [95]	3-D U-net	PET/CT derived images	100	66±11	Accuracy	79%	87%	75%	79%
Lesserman *et al.*	2018 [74]	Double CNN	Low-dose chest CT	1744	55 to 74*	Sensitivity	93 %^$^		72 %	92 %
Sartoretti *et al.*	2023 [85]	3-D U-net	ECG-gated CT	56	63±9	ICC	0.64	0.95	0.93	0.99
Takahashi *et al.*	2023 [123]	3-D U-net	ECG-gated CT	1369^#^	63±13 (500 pts) 66±12 (409 pts) 60±10 (400 pts)	Pearson’s correlation	0.85	0.98	0.98	0.99

*: age range; ^#^: 60 scans were synthetically generated using a generative adversarial network from coronary CT angiography; ^$^: as per Lesserman et al.[74], a single sensitivity for LM and LAD is reported, thereby hindering the possibility of distinguishing vessel-specific sensitivity values; *CACS* coronary artery calcium score, *CNN* convolutional neural network, *CT* computed tomography, *ECG* electrocardiography, *ICC* intraclass-correlation coefficient, *LAD* left anterior descending coronary artery, *LCx* left circumflex coronary artery, *LM* left main trunk, *PET* positron emission tomography, *pts* patients, *RCA* right coronary artery, *SD* standard deviation

### Computational time

**Fig. 4:**
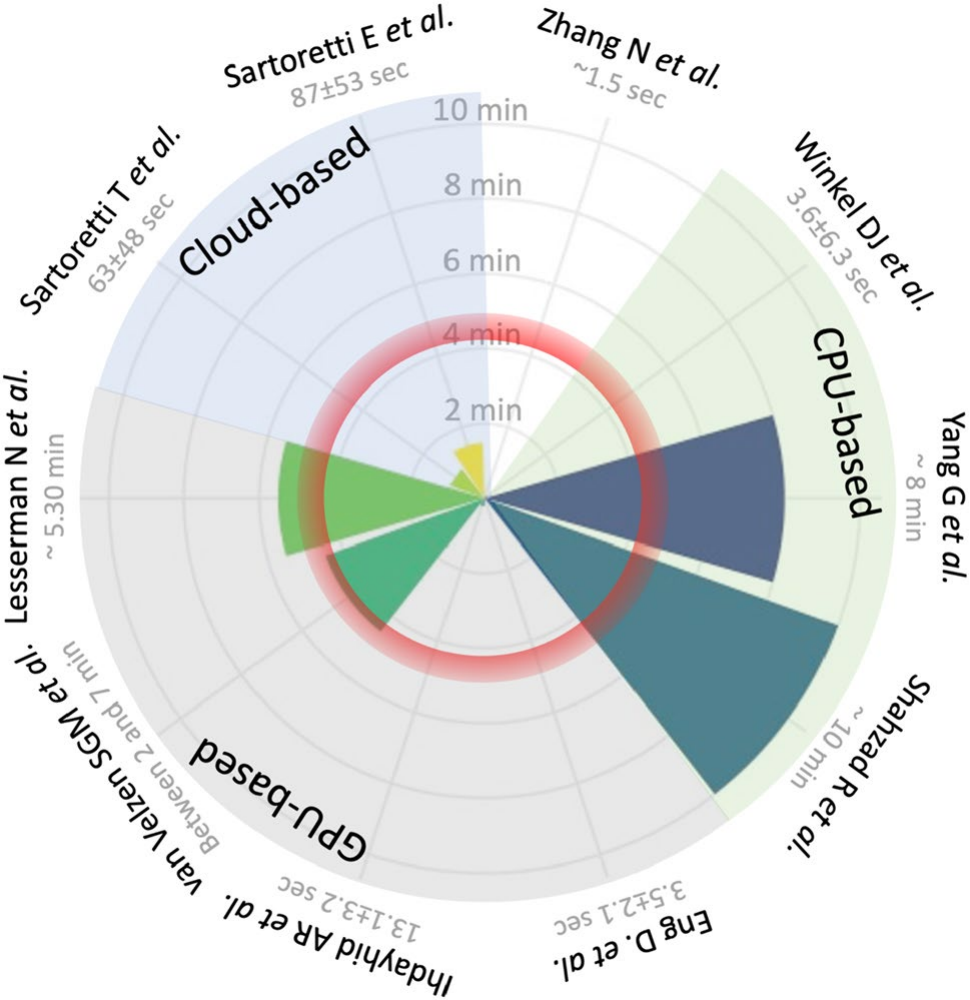
Computational time taken to quantify CACS according to different technical set-ups. The computational time taken to analyze the images was extremely heterogeneous between studies, varying from a few seconds (invisible cones) to approximately ten minutes. This heterogeneity was highly dependent on the computational approach used. However, most studies reported computational times lower than that taken by experts (red ring). The latter was based on the results by Eng *et al.* [83] *CPU* central processing unit, *GPU* graphics processing unit, *min* minutes, *sec* seconds

Algorithm architecture, use of graphic processing unit, and the number of cores of the computer processing unit strongly impacted the computational time taken to quantify CACS, generating heterogeneous results (mean computational time: 3 min, range: 2 s to 10 min, Fig. 4) [74, 81, 83–86, 96, 98–100]. Irrespective of the computational time, these results show that automatizing CACS calculation may reduce its costs and streamline the workflow of imaging departments.

### Deep learning image reconstruction

The growing awareness of the potential risks associated with ionizing radiation usage forced CT manufacturers and the medical community to adopt low-dose protocols [101]. Low-dose images inherently exhibit higher image noise and artifacts than standard-dose images. Hence, in the last five years, vendors have introduced deep learning reconstruction (DLR) to address these problems [102, 103]. To date, three DLR methods have been developed: *TrueFidelity*, *AiCE*, and *Precise Image*. These algorithms differ based on the input data used in the training phase, the framework they were built on, and the ground truths adopted to compare their performances [102]. Inputs included raw data (sinograms), filtered back projections (FBP), or iterative reconstruction IR images (specifically model-based iterative reconstruction), while frameworks distinguished between direct and indirect. Direct frameworks generated DLR-optimized images in a single step by applying DL algorithms to sinograms (*TrueFidelity* and *Precise Image*). Indirect frameworks either generate DL-optimized sinograms before reconstructing images or optimize reconstructed images using DL algorithms (*AiCE*) [102]. A more comprehensive and detailed description of the strategies used to create DLR can be found in Koetzier et al. [102].

**Fig. 5:**
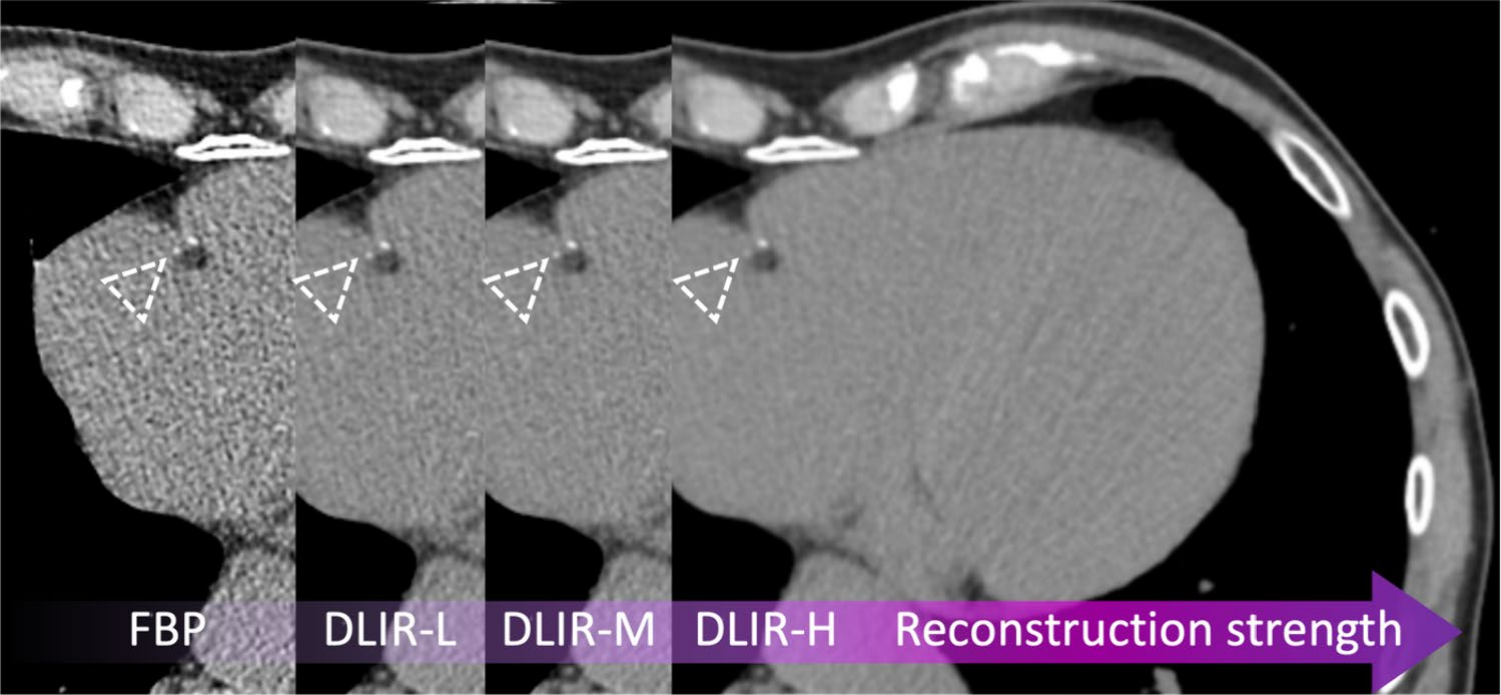
CAC detectability according to different image reconstruction algorithms. Coronary artery calcium detectability according to different image reconstruction algorithms in a 78-year-old hypertensive male. Two small calcifications were detectable on the filtered back projection image (dotted white arrowhead) along the course of the right coronary artery. However, the same calcifications (dotted white arrowhead) on the corresponding images, reconstructed using various deep-learning strengths, were less evident. Specifically, using the highest deep-learning reconstruction strength (DLIR-H), the margins of the bigger calcification were more blurred, while the smaller classification became barely evident. The Agatston score reduced from 691 to 688, 674, and 667, with FBP, DLIR-L, DLIR-M, and DLIR-H, respectively. *CAC* coronary artery calcium, *DLIR* deep-learning image reconstruction, *FBP* filtered back projections

The use of DLR led to a reduction in image noise and a concomitant increase in SNR and CNR compared to FBP [104–106]. However, a phantom study using standard acquisition parameters showed that, compared with other image reconstruction techniques, DLR failed to detect calcifications ≤ 1.2 mm [107]. While most studies showed Agatston scores being comparable throughout different reconstruction techniques, its values were reduced with increasing DLR strengths compared to FBP [104–106]. This translated into a downward reclassification of risk scores in 2 to 8% of patients [104–107]. The sole study comparing 3 mm FBP-reconstructed ECG-gated images to 1 mm, low-dose, non-gated DLR-reconstructed images showed that the latter underestimated the CACS (94 ± 249 vs. 105 ± 249) and had 90% accuracy in classifying different risk classes (Fig. 5) [108]. These results prove that DLRs are a promising tool. However, their intrinsic tendency to down-quantify the Agatston score may profoundly impact treatment strategies. Hence, their implementation in CACS evaluation needs additional studies or correction factors.

### Extracardiac findings

Although CACS images rely on a limited field-of-view, a holistic AI algorithm aiming to automatize CACS reporting should not overlook possible extracardiac findings. A systematic review including more than 11,000 patients undergoing cardiac CT showed that 41% of them had extracardiac findings (ECFs) with an average prevalence of clinically significant ECFs of 16% [109]. Although these results may not be entirely transferable to patients undergoing CACS, they highlight the need for additional automated AI-based evaluation of ECFs. Suspicious lung nodules, hiatal hernia, emphysema, enlarged lymph nodes, and pleural effusion accounted for 63% of clinically significant ECFs [109] and are readily diagnosable on non-enhanced CT images. The sole study exploring the automated detection of CAC and solid lung nodules on low-dose CT images had ambiguous results [110]. The AI-to-expert agreement was excellent in discriminating between patients with CACS 0 and those with CACS > 0 (k = 0.85), whereas the performance of nodule detection was suboptimal (k = 0.42) [110]. These results prove that further research is needed to improve the performance of AI algorithms in handling multiple inputs.

## Conclusions

The discrepancies between the number of chest CT (~ 12,7 million), PET/CT (~ 1,8 million), and ECG-gated CACS scans (~ 57,500) acquired in the United States [111] highlight the potential to diagnose/refine the ASCVD risk in a wider population [112]. However, it also confirms the future additional diagnostic burden expected to impact the medical imagers community. In this context, AI may be a valuable tool to alleviate the workload, supporting everyday routine. While this process is expected to transform medical imaging, it will likely not put imagers out of their job nor alter their professional identity or autonomy [11, 113, 114]. Instead, it will enable them to profit from the human-to-AI relationship, benefiting from complementary strengths, ultimately fortifying the central role that imaging plays in modern medicine. Before this becomes a reality, additional steps must be taken into consideration. Automated CACS quantification needs to be a reliable, error-free, and easily implementable tool, irrespective of the computational power. An interesting perspective to further improve algorithms’ performances would be gathering a highly curates, analyzed CACS dataset, including scans acquired from different vendors, serving as the global benchmark to test algorithms. Additionally, testing fully trained algorithms on different datasets would ensure the reproducibility of the results. The effects of image filters and reconstruction on the algorithm’s performances should be clarified in detail. DLR could be retrained using ECG-gated, normal-dose scans, enabling the quantification of the performance changes compared with non-dedicated training. Finally, a holistic approach to image analysis should be regarded as the ultimate goal, including the evaluation of extracardiac findings, bone density, and anemia detection, quantifying blood density [115], ensuring to derive the most from a single scan.

## Data Availability

No datasets were generated or analysed during the current study.
